# miRNAs for the Detection of MultiDrug Resistance: Overview and Perspectives

**DOI:** 10.3390/molecules19055611

**Published:** 2014-04-30

**Authors:** Andreas Gisel, Mirna Valvano, Imane Ghafir El Idrissi, Patrizia Nardulli, Amalia Azzariti, Antonio Carrieri, Marialessandra Contino, Nicola Antonio Colabufo

**Affiliations:** 1 Institute for Biomedical Technologies, CNR Bioinformatics and Genomics Research Group, Via Amendola 122/D, Bari 70126, Italy; E-Mails: andreas.gisel@ba.itb.cnr.it (A.G.); mirna.valvano@libero.it (M.V.); 2 Biofordrug srl, Università degli Studi di Bari, via Orabona 4, Bari 70125, Italy; E-Mail: gha_imane@yahoo.it; 3 Clinical and Preclinical Pharmacology Laboratory, National Cancer Research Centre, Istituto Tumori Giovanni Paolo II, Viale O. Flacco, 65, Bari 70124, Italy; E-Mails: p.nardulli@oncologico.bari.it (P.N.); a.azzariti@oncologico.bari.it (A.A.); 4 Università degli Studi di Bari “Aldo Moro”, Via Orabona 4, Bari 70125, Italy; E-Mails: antonio.carrieri@uniba.it (A.C.); marialessandra.contino@uniba.it (M.C.)

**Keywords:** microRNAs, MDR, early diagnosis, MDCK-MDR1, P-glycoprotein

## Abstract

The goal of the present paper is to establish and validate the link between cancer diagnosis and therapy by microRNAs detection. The induction *in vitro* of some specific microRNAs after treatment with MDR ligands has been outlined. Starting from the results obtained by *in vitro* induction of MDCK and MDCK-MDR1 cells treated by a MDR1 ligand, a new scenario in the early diagnosis and chemotherapy could be disclosed. To corroborate this perspective a short overview on pancreatic cancer diagnosis and chemotherapeutic treatment has been reported.

## 1. Introduction

MultiDrug Resistance (MDR) is the major obstacle to a successful cancer therapy and is often associated with an increased efflux of anticancer drugs due to some proteins belonging to the ATP-Binding Cassette (ABC) transporters family such as P-gp, BCRP and MRP1 [1,2,3,4,5]. P-gp, exerting a physiological activity as first line of defense of our body and together with protein ABCG2/BCRP, is localized at the apical level in cells membranes of different cellular compartments such as liver, intestine, kidney, placenta [1,2,3,4,5]. This strategic localization gives P-gp a crucial role as responsible for drugs absorption and accumulation. MRP1 (ABCC1) also belonging to ABC transporters, localized at the basolateral level in cells membranes, facilitates the transport of the substrates in the blood [1,2,3,4,5].

The involvement of P-gp, BCRP and MRP1 in MDR is due to their overexpression in resistant tumor cells [2]. In particular MDR1/P-gp behaves as “protein scavenger” able to “capture” drugs and transport them out of cells.

MicroRNAs (miRNAs) are small endogenous non-coding RNAs responsible for the post-transcriptional regulation of target genes by the interaction with specific sequences in their 3' untranslated region (3'UTR), leading to mRNA degradation and/or translational inhibition [6]. MiRNAs play important roles in many cellular processes such as proliferation, apoptosis and differentiation, and in many physiological processes such as metabolism, cardiogenesis, development and function of the nervous and immune systems [6,7,8]. The miRNAs dysregulation in cancers are widely reported and recent literatures revealed a correlation of miRNAs levels in biological fluids with chemotherapy response [8]. To *et al.* reported a double regulation of MDR, direct and indirect, mediated by miRNAs [8]. Moreover, a recent study demonstrated that a miRNAs dysregulation and an altered expression of Dicer and Argonaute, enzymes involved in miRNA maturation process, have been found in a doxorubicin-resistant breast cancer cell line, MCF-7/DOX [9]. Several miRNAs have been found to regulate drug resistance genes such as ABCG2 [10,11,12,13] and MDR1 [9,14] and the modulation of miRNAs expression or function has been reported to alter the sensitivity of cancer cells to anticancer drugs [15,16].

MiR-27a and miR-451 are involved in the activation of MDR1 expression [14], and in particular miR-27a has been found able to modulate MDR1 expression by the inhibition of FZD7/β-catenin pathway in hepatocellular carcinoma cells [17]. MiR-298 has been found able to increase MDR1 expression and so responsible for doxorubicin-resistance of metastatic breast cancer [18].

The hypothesis that miRNAs could be involved in the modulation of P-gp expression is supported by suggestive evidence: (i) miR-27a and miR-451 induce an increased P-gp expression in ovarian cancer cells [9,14], (ii) miR-451 also down-regulates MDR1 expression in MCF7/DOX-resistant cells [9]; (iii) changes in miR-381 and miR-495 expression are inversely correlated to MDR1 expression and the development of MDR [19].

In the first part of this paper, we monitored the induction of some specific miRNAs by the treatment of wild type cell line (MDCK cells) and in the corresponding cell line where P-gp is overexpressed (MDCK-MDR1 cells) unsensitive to antineoplastic treatment. In particular we stimulated MDCK cells with a specific P-gp substrate **8c** and a selective P-gp inhibitor **8a**, as reported in Table 1 [20], verifying the down- or up-regulation of miRNAs with respect to untreated cells.

**Table 1:** Biological evaluation of tariquidar derivatives **8a** and **8c**.

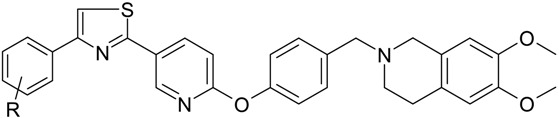				
**Compound**	**R**	**MDR1 ^a^ EC_50_, µM**	**P_app_^a^**	**MDR1 interacting mechanism ^a^**
**8a**	3-OH	**0.25 ± 0.02**	0.9	*inhibitor*
**8c**	4-NO_2_	**0.51 ± 0.032**	2.7	*substrate*

^a^ All data are reported in reference [20].

Preliminary eight microRNAs have been identified as highly expressed in MDCK-wild type and are also reported in many human tumors as glioblastoma, bladder cancer, breast cancer, renal cancer as reported in the literature [12,13,14]. Aims of this study are: (i) to probe a link between some miRNAs and MDR P-gp-mediated; (ii) to improve the knowledge of the pathways involved in cancers characterized by MDR; (iii) to verify if different miRNAs are modulated by P-gp inhibitor and substrate; (iv) to suggest some miRNAs as probes to diagnose MDR and to address chemotherapy.

## 2. Results and Discussion

Eight microRNAs have been identified as highly expressed in MDCK-wild type and MDCK-MDR1 as listed in Table 2.

**Table 2:** Frequence of miRNAs higly expressed in MDCK-wt and MDCK-MDR1.

miR-Dog	miR-Homo	Freq. MDCK-wt	RPM Freq. MDCK-wt	Freq. MDCK-MDR1	RPM Freq. MDCK-MDR1
cfa-miR-182	**hsa-miR-182-5p**	835,232	32,507,727	785,269	44,147.288
cfa-miR-21	**hsa-miR-21-5p**	767,174	29,858.950	582,432	32,744.188
cfa-miR-10a	**hsa-miR-10a-5p**	1,610,023	62,662.126	944,420	53,094.446
cfa-miR-27b	**hsa-miR-27a-3p**	612,930	23,855.867	319,758	17,977.170
cfa-miR-30a	**hsa-miR-30a-5p**	515,654	20,069.943	312,771	17,584.374
cfa-miR-26a	**hsa-miR-26a-5p**	545,522	21,232.388	359,038	20,185.414
cfa-miR-10b	**hsa-miR-10a-5p**	2,127,806	82,813.929	1,602,329	90,080.803
cfa-miR-191	**hsa-miR-191-5p**	636,561	24,775.571	376,207	21,150.622

Freq: Frequence for each miRNA higly expressed; RPM Freq: normalization of frequence for each miRNA higly expressed.

These miRNAs represent the background and so they cannot be considered for our studies. By contrast, the expression of five miRNAs, modulated only by compound **8c** displaying MDR substrate activity, has been detected in MDCK-wt and MCDK-MDR1 treated with **8c**. As expected, P-gp inhibitor **8a** did not exert any effect on miRNAs expression because it has been tested as a negative control. Cfa-miR-181d (hsa-miR-181a-5p), cfa-miR-218 (hsa-miR-218-5p) were found up-regulated in **8c**-treated MDCK-MDR1 cells with respect to untreated MDCK-MDR1 cells, while cfa-miR-454 (hsa-miR-130a-3p), cfa-miR-424 (hsa-miR-424-3p), cfa- miR-1307 were found down-regulated, as depicted in Table 3.

**Table 3:** Expression of five microRNAs known and selected by miRDeep2 in MDR, regulated by compound **8c**.

miR-Dog	miR-Homo	RPM-MDCK-MDR1	RPM–Substrate-8c-MDCK-MDR1	Substrate-8c-MDCK-MDR1/MDCK-wt
cfa-miR-181d	**hsa-miR-181a-5p**	0.000	122.319	6.93450378
cfa-miR-218	**hsa-miR-218-5p**	8.814	40.113	2.186153174
cfa-miR-454	**hsa-miR-130a-3p**	9.264	0.000	−3.211643438
cfa-miR-424	**hsa-miR-424-3p**	11.963	0.000	−3.580449275
cfa-miR-1307	-	31.864	0.000	−4.993842191

### 2.1. MiRNAs and MDR Pathway

#### 2.1.1. cfa-miR-424 (hsa-miR-424-3p)

MiRNA-424, identified as down-regulated in **8c**-treated MDR1-MDCK cells, is involved in different pathways leading to MDR up-regulation. In particular, it has been reported that miR-424 mediates the induction of the hypoxia inducing factors HIF-1α and HIF-2α [21]. HIF-1α is responsible for MDR1 up-regulation. Moreover, miR-424 modulates also the expression of protein cullin 2 (CUL2), a scaffolding protein displaying a pivotal role in the assembly of the ubiquitin ligase system, thereby stabilizing HIF-1α [21]. Considering these pathways, compound **8c** inducing a miR-424 down-regulation could lead to a consequent reduction of HIF1α and consequently of MDR P-gp-mediated.

#### 2.1.2. cfa-miR-454 (hsa-miR-130a-3p)

miR-130a-3p (corresponding to miR-454 in the dog) expression in humans is reduced in response to the action of the substrate **8c**, as reported in Table 3. A recent study identified the miRNAs differentially expressed in a tumor ovarian carcinoma cell line (SKOV3) and in the parental cell line insensitive to cisplatin (SKOV3/CIS). This different miRNAs profile suggests that these miRNAs and their target proteins could play a crucial role in the development of cisplatin resistance in ovarian cancer [22]. The expression levels of miR-130a were higher in SKOV3/CIS cells than in SKOV3 cells; also P-gp expression level was very high in SKOV3/CIS and absent in SKOV3 cells. Overall, these results demonstrated that miR-130a up-regulation in resistant ovarian cells was correlated to MDR1-mediated cisplatin resistance. The MDR induction is probably due to a direct modulation of PI3K /Akt, PTEN and mTOR, by miR-130a-3p up-regulation [22].

Another study by Xu *et al.* demonstrated that the up-regulation of miR-130a, induced by cisplatin, leads to cisplatin resistance by the inhibition of RUNX3 and the activation of Wnt signaling [23]. Therefore, also in this case the down-regulation of miR-130a could lead to a decrese of MDR.

#### 2.1.3. cfa-miR-218 (hsa-miR-218-5p)

MiR-218-5p was detected as up-regulated in **8c**-treated cells; its down-regulation is reported in etoposide resistant MCF7VP breast cancer cell line compared to MCF7 wild type [24]. This effect has been considered an indicator of etoposide-resistance in this cell line since it is also accompanied to an increased expression of ABCC1 and ABCC6. Therefore, its down-regulation by MDR1 substrates could lead to a decreased expression of MDR gene.

#### 2.1.4. miR-181a-5p

MiR-181a has been considered as tumor suppressor in human acute monocytic leukemia (AML) and its overexpression has been demonstrated to induce apoptosis of AML blasts [25,26,27]. This effect is probably linked to the modulation of the expression of the antiapoptotic protein BCL2. Apoptosis induced by the antineoplastic agent, daunorubicin (DNR), can be blocked by BCL-2 overexpression and so the suppression of BCL-2 expression enhance DNR-induced apoptosis [28]. BCL-2 has been identified in several studies as one of the potential targets of miR-181a and so the down-regulation of miR-181a induced in K562 cells resistance to DNR and the up-regulation of miR-181 sensitized the K562/A02 cells to DNR. Therefore, miR-181a might sensitize K562/A02 cells to DNR by repressing the BCL-2 protein expression [27].

#### 2.1.5. miR-1307

cfa-miR-1307 identified by the program miRDeep2 in MDR1-MDCK cells treated with the substrate **8c**, do not correspond to any human miRNA, and it is probably unknown. However, this miRNA is down regulated in the MDR1-MDCK cells treated with the substrate **8c** compared to the parental MDCK wild type cells. Moreover, TargetScanHuman (a software for the prediction of microRNA targets) identified as probable cfa-miR-1307 target, the BCL2 gene, responsible for the corresponding anti-apoptotic protein.

### 2.2. Perspective of microRNAs in MDR Diagnosis

Several studies have demonstrated that some miRNAs are associated with tumor promotion (oncogenes) or could inhibit tumors by reducing cell proliferation, cell survival (tumor suppressors) and cellular differentiation; thus, restoring the expression of some miRNAs in tumor cells could lead to differentiation and induce malignant cells into a normal state.

Recently, the deregulation of miRNA expression in pancreatic cancer tumors has been reported [29,30,31,32,33,34,35] and the monitoring of this deregulation has been performed at different stages of cancer. It has already reported that miRNAs expression varied in different solid tumors (breast, colon, lung, pancreas, prostate, and stomach) giving information on the response of cancer cells to different treatment (Table 4). For example, a study, performed on the human pancreatic cancer cell line BxPC-3, demonstrated that the inhibition of proliferation induced by trichostatin A (TSA) was time and dose-dependent and this effect was accompanied by a different miRNAs expression profile [36]; TSA treatment induced the expression of tumor suppressor miR-200c and miR-21 leading to growth arrest. Similarly, curcumin induced up-regulation of miR-22 and down-regulation of miR-199a in human pancreatic cells [30], 5-aza-2'-deoxycytidine exposure induced the up-regulation of several miRNAs (miR-107, miR-103, miR-29a, miR29b and miR-320) in MiaPACA-2 and Panc-1 cell lines [37]. Moreover, the natural treatment with 3,3-diindolylmethane (DIM) or isoflavone induced the down-regulation of miR-200b, miR-200c, let-7b, let-7c, let-7d, and let-7e in gemcitabine-resistant cells [38].

**Table 4:** Panel of microRNAs in normal and pancreatic cancer.

microRNA	Normal tissue	Pancreatic cancer	PDAC
miR-103	Basal level	Up-regulated	Basal level
miR-107	Basal level	Up-regulated	Basal level
miR-155	Basal level	Down-regulated	Basal level
miR-216	Basal level	Basal level	High level
miR-217	Basal level	Basal level	High level
miR-133a	Basal level	Basal level	Absent
miR-99	Not detected	Detected	Detected
miR-100	Not detected	Detected	Detected
Mir-100-1,2	Not detected	Detected	Detected
miR-125a	Not detected	Detected	Detected
miR-125b-1	Not detected	Detected	Detected
miR199a-1	Not detected	Detected	Detected
miR199a-2	Not detected	Detected	Detected
miR-21	Basal level	Detected	Detected
miR-221	Not detected	Detected	Detected
miR-222	Not detected	Detected	Detected
miR-181a	Not detected	Detected	Detected
miR-181b	Not detected	Detected	Detected
miR-181d	Not detected	Detected	Detected
miR-196a	Basal level	Detected	Detected

The miRNA expression pattern may discriminate normal pancreas from tumors; indeed, the up-regulation of miR-103 and miR-107, and the down-regulation of miR-155 can distinguish tumors from the normal pancreas [37]. Moreover, the presence of miR-216 and -217 and the lack of miR-133a expression was a characteristic feature of the pancreatic tissue.

This different expression profile can be useful for miRNAs-based diagnosis [34]. The up-regulation of miR-99, miR-100, miR-100-1/2, miR-125a, miR-125b-1, miR-199a-1, and miR-199a-2 was detected in cancers and in chronic pancreatitis but not in normal pancreatic tissue; the up-regulation of miR-21, miR-221, miR-222, miR-181a, miR-181b, miR-181d, and miR-155 was found in tumors compared to benign pancreatic tissue [32,33]. Moreover, the up-regulation of miR-21 was predictive of a poorer outcome and may be an important biomarker for prognosis of pancreatic cancer [39]. MiR-196a and miR-217 can discriminate between healthy tissue, PDAC, and chronic pancreatitis [40]. The expression of miR-21, miR-210, miR-155, and miR-196a in the plasma of patients revealed the cancer and among them overexpression of miR-155 and miR196a is due to the progression of the pathology. Therefore, the identification of specific miRNAs in plasma can provide sensitive and specific blood-based biomarkers for pancreatic cancer diagnosis [41]. Moreover, the high levels of miR-155, miR-203, miR-210 and miR-222 in pancreatic tumors were also associated with increased risk of death compared to patients with tumors with low expression of the same miRNAs [42].

### 2.3. Usefulness of miRNAs Recognition in Multidrug Resistance Chemotherapy: On Overview on Pancreatic Cancer

To date, there are different therapeutic approaches for pancreatic cancer, including single or multi-agents chemotherapy, chemotherapy followed by chemoradiation, or immediate concurrent chemoradiation. In patients affected by pancreatic metastatic cancer, the overall survival is of 6 months from the time of diagnosis. Gemcitabine has been the standard treatment (from 1997). Recently, the combination of the antineoplastic agents 5-fluorouracil, oxaliplatin, irinotecan and leucovorin (FOLFIRINOX) have been used as alternative strategy [43] leading to an improved response of advanced metastatic disease compared with gemcitabine alone and other historical treatments [44,45,46,47,48].

Folfirinox showed a median overall survival (11 months) higher than that of gemcitabine (6.8 months) and a median progression-free survival almost doubled (6.4 months) with respect to gemcitabine (3.3 months) [49]. However, folfirinox treatment in some clinical trials has showed high toxicity with respect to gemcitabine regimen (neutropenia, diarrhea, fatigue) [49].

A relationship between the expression of some miRNAs and survival of patients with pancreatic adenocarcinoma has been already reported and miR-21, miR-155, miR-203, miR-210 and miR-222 have been identified as potential predictors of survival [42,50].

The expression of miR-155, miR-203, miR-210, miR-216, miR-217 and miR-222, differentially expressed in pancreatic tumors compared to normal tissues, has showed an high correlation with the overall survival. In particular, patients with elevated expression of all four miRNAs had a 6.2-fold increased risk of tumor-related death with respect to patients with a lower expression of these miRNAs [51].

High miR-21 expression has been detected in patients with a significantly shorter overall survival (OS) [50] and a correlation between miR-21 expression and gemcitabine resistance has been proved. Also miR-200 is involved in chemosensitivity of pancreatic cancer cells and its decreased expression together with an increased expression of miR-21 are linked to gemcitabine resistance in pancreatic cancer cell lines [51].

Therefore, since miR-21 is expressed in all pancreatic adenocarcinoma forms and it is significantly up-regulated after gemcitabine treatment, miR-21 could be a prognostic factor leading pancreatic cancer therapy [50].

## 3. Experimental

### 3.1. Cells Lines

*Materials:* Cell culture reagents were purchased from Celbio s.r.l. (Milano, Italy).

*Cell cultures* MDCK-MDR1 and MDCK cells are a gift of P. Borst, NKI-AVL Institute, Amsterdam, Nederland. All MDCK cells were grown in DMEM high glucose supplemented with 10% fetal bovine serum, 2 mM glutamine, 100 U/mL penicillin, 100 µg/mL streptomycin, in a humidified incubator at 37 °C with a 5% CO_2_ atmosphere. MDCK cells were cultured to 80% confluence, the medium removed and the cells rinsed in PBS. The cells were scraped and suspended in PBS, then centrifuged at 1500 rpm for 15 min. The supernatant was discarded and the pellet used for the analysis.

*Treatment with* **8a** *and* **8c**. MDCK-MDR1 and MDCK cells were treated with 0.5 µM **8a** and **8c** (solved in medium buffer) at 48h of exposure. The concentration has been selected taking into account the activity values (EC_50_ = 0.25 and 0.51 µM, respectively) of the two compounds towards P-gp [20]. The exposure time has been determined considering the induction of involved pathways into the cells

### 3.2. Data Processing

In a first step of this work, we used indexed genome of dog to map reads. In this operation miRdeep2 used an algorithm which divided sequences that map to those that do not map to the genome of dog, so we discarded sequences that don’t map on genome of dog. In the second step we used a script of miRDeep2 able to extract all the sequences including the respective name given in miRBase [52] in relation to the human species and the species of the dog, so as to compare them in the next step. For this step we have needed of a script in perl that extracts microRNA with their name for each species from miRBase. So we have obtained a file for human, a file for dog and a file for the other species. So in the next phase miRDeep2 used these files of reference and compared, previously selected reads, with sequences having a distinct name miR extracted from miRBase. So it is possible to assign a name to each sequence as compared with miR of man and dog shows in miRBase. The final phase includes the acquisition of two files for each experiment, the first file contains all the known sequences of miR of dog with same “seed” in human and the second file contains unknown miR for dog and for other species. But we focused on known miR that have a name in miRBase both for dog that for human because characterized by same seed. We have obtained these outputs: (1) miRNAs known for MDCK-wt and MDCK-MDR1 (2) miRNAs known for each cell line treated respectively with the compound **8a** (MDR1-inhibitor) and 8c (MDR1-substrate). We filtered known miRNAs according to high score to eliminate repeated sequences and a shorter sequence of the same microRNA. The next step was to compare all the miRNAs obtained in each experiment with the source file in which we have calculated the frequency with which each sequence of microRNA is expressed in each experiment. At this point we have created a script in perl to calculate a “fold change” between:

(i) MDCK- MDR1 *vs.* MDCK-wt; (ii) MDCK-MDR1 treated with compound 8a and 8c *vs.* untreated MDCK- MDR1. “Fold change” is the ratio between the expression of miRNAs in each cell line and its control, where the control is represented by MDCK-wt or MDCK-overexpressing MDR1 (MDCK-MDR1) (See Tables S2 and S3 in supplementary materials).

## 4. Conclusions

In this article the role of miRNAs in MDR has been pointed out. For this purpose, an brief experimental section has been carried out where the link between some miRNAs and the induced MDR has been demonstrated. This finding confirmed that miRNAs could be routinely employed both for the diagnosis and to drive the pharmacological treatment since the experimental section demonstrated that the over-and/or down-expression of miRNAs can be drug-sensitive. This preliminary study aims to hit both the diagnostic and therapeutic items, and it could drive the chemotherapeutic strategy taking into account the level of drug-sensitive miRNAs. To corroborate this perspective a short overview on pancreatic cancer diagnosis and chemotherapeutic treatment has been reported.

## Supplementary Materials

Supplementary materials can be accessed at: http://www.mdpi.com/1420-3049/19/5/5611/s1.
