# Supplementary material for: miRNAs for the Detection of MultiDrug Resistance: Overview and Perspectives
Source: Molecules. 2014 Apr 30;19(5):5611–23. doi: 10.3390/molecules19055611 (PMC6270919; doi:10.3390/molecules19055611)
Supplement: Supplementary file 1 [file molecules-19-05611-s001.pdf]

Supplementary Materials

Bioinformatics

Data Filtering

Taking advantage of the scripting language perl, the first operation performed on RNA sequences obtained from Illumina (raw data), has been to identify the “barcode” or the short nucleotide sequence added to the RNA sequences for each experiment, to differentiate the reads and to extract only RNA sequences. In the second step of filtering the raw data, we carried out the removal of adapters from the ends of the sequences. Finally we filtered the data obtained according to the length of RNA sequences, extracting only sequences presenting a length of between 18–26 nucleotides, corresponding to the typical measure of microRNAs. So we started from a number of sequences equal to 55,744,311 for the samples of MDCK cells overexpressing MDR1 and treated with the compound **8c**, to obtain a number of sequences 38,152,847 characterized by a length of 18–26 nucleotides. In the presence of compound **8a** we started from a number of sequences extracted amounted to 44,324,719, to arrive at a number of sequences sorted equal to 26,742,105. The same filtering operation was performed on cell lines MDCK overexpressing MDR1 without treatment and on MDCK wild type cells, as seen in Table 5.

Table S1. Data raw statistics (Data Absolute Number Seq). Filtering of sequences 18–30 nucleotides (Data 18–30 Number Seq), % of sequences with length 18–30, number of redundant sequence with length of 22 nucleotides (Data Absolute Number Seq 22 mer), % of sequences with length 22 nucleotides. This length of 22 nucleotides is overexpressed for microRNA present in these data.

Name Experiment	Data Absolute Number Seq	Data 18–30 Number Seq	%Seq 18–30	Data Absolute Number Seq 22 mer	%Seq 22 mer
Substrate-8c-MDR1	55,744,311	38,152,847	6,844	13,211,228	2,369
Inhibitor-8a-MDR1	44,324,719	26,742,105	6,033	10,267,265	2,316
MDCK-MDR1	29,706,985	17,828,639	6,001	6,944,168	2,337
MDCK-wt	42,287,302	25,755,368	6,090	10,292,278	2,43

The reads extracted and having a length between 18–26 nucleotides corresponding to the typical measure of microRNAs, were subjected to miRDeep2. The length of microRNA found more is 22 nucleotides (Data Absolute Number Seq 22 mer) as shown in Table I. MiRDeep2 is an algorithm that can identify microRNA canonical and non-canonical, then transposons derived from elements in the DNA of the species concerned. miRDeep2 is able to identify microRNAs with an accuracy ranging from 98.6% to 99.9% and it is based on the miRNA biogenesis model, it aligns reads to potential hairpin structures in a manner consistent with Dicer processing, and assigns scores that represent the probability that hairpins are true miRNA precursors. In addition to the detecting of known and novel miRNAs, miRDeep2 estimates also their abundance.

Table S2. Expression of miRNAs known and selected by miRDeep2, presents in Multidrug resistance with basal level.

miR-Dog	miR-Homo	MDCK-MDR1/MDCK-wt	Substrate-8c-MDCK-MDR1	Inhibitor-8a-MDCK-MDR1
cfa-miR-93	**hsa-miR-17-5p**	Equal	Equal	Equal
cfa-miR-196a	**hsa-miR-196a-5p**	Equal	Equal	Equal
cfa-miR-130b	**hsa-miR-130a-3p**	Equal	Equal	Equal
cfa-miR-628	**hsa-miR-628-5p**	Equal	Equal	Equal
cfa-miR-19b	**hsa-miR-19a-3p**	Equal	Equal	Equal
cfa-miR-345	**hsa-miR-345-5p**	Equal	Equal	Equal
cfa-miR-185	**hsa-miR-185-5p**	Equal	Equal	Equal
cfa-miR-421	**hsa-miR-421**	Equal	Equal	Equal
cfa-miR-18a	**hsa-miR-18a-5p**	Equal	Equal	Equal
cfa-miR-1839	**hsa-miR-3927-3p**	Equal	Equal	Equal
cfa-miR-193b	**hsa-miR-193a-3p**	Equal	Equal	Equal
cfa-miR-193a	**hsa-miR-193a-5p**	Equal	Equal	Equal
cfa-let-7c	**hsa-let-7a-5p**	Equal	Equal	Equal
cfa-miR-184	**hsa-miR-184**	Equal	Equal	Equal
cfa-miR-676	-	Equal	Equal	Equal
cfa-miR-1306	**hsa-miR-1306-5p**	Equal	Equal	Equal
cfa-miR-574	**hsa-miR-574-3p**	Equal	Equal	Equal
cfa-miR-497	**hsa-miR-15a-5p**	Equal	Equal	Equal
cfa-miR-204	**hsa-miR-204-5p**	Equal	Equal	Equal
cfa-miR-30a	**hsa-miR-30a-5p**	Equal	Equal	Equal
cfa-miR-107	**hsa-miR-103a-3p**	Equal	Equal	Equal
cfa-miR-219-5p	**hsa-miR-219-1-3p**	Equal	Equal	Equal
cfa-miR-197	**hsa-miR-197-3p**	Equal	Equal	Equal
cfa-miR-152	-	Equal	Equal	Equal
cfa-miR-1271	**hsa-miR-96-5p**	Equal	Equal	Equal
cfa-miR-126	**hsa-miR-126-5p**	Equal	Equal	Equal
cfa-miR-328	**hsa-miR-328**	Equal	Equal	Equal
cfa-miR-149	**hsa-miR-149-5p**	Equal	Equal	Equal
cfa-miR-503	-	Equal	Equal	Equal
cfa-miR-146a	**hsa-miR-146a-5p**	Equal	Equal	Equal
cfa-miR-138a	**hsa-miR-138-5p**	Equal	Equal	Equal
cfa-miR-375	**hsa-miR-375**	Equal	Equal	Equal
cfa-miR-195	**hsa-miR-15a-5p**	Equal	Equal	Equal
cfa-miR-660	**hsa-miR-660-5p**	Equal	Equal	Equal
cfa-miR-23b	**hsa-miR-23a-3p**	Equal	Equal	Equal

Table S3. Equal expression of miR between MDCK-MDR1 and MDCK-wt. "Equal" means equal expression of miR.

miR-Dog	miR-Homo	MDCK-MDR1-MDCK-wt
cfa-miR-93	**hsa-miR-17-5p**	Equal
cfa-miR-196a	**hsa-miR-196a-5p**	Equal
cfa-miR-130b	**hsa-miR-130a-3p**	Equal
cfa-miR-628	**hsa-miR-628-5p**	Equal
cfa-miR-19b	**hsa-miR-19a-3p**	Equal
cfa-miR-424	**hsa-miR-424-3p**	Equal
cfa-miR-181d	**hsa-miR-181a-5p**	Equal
cfa-miR-345	**hsa-miR-345-5p**	Equal
cfa-miR-185	**hsa-miR-185-5p**	Equal
cfa-miR-421	**hsa-miR-421**	Equal
cfa-miR-18a	**hsa-miR-18a-5p**	Equal
cfa-miR-1839	**hsa-miR-3927-3p**	Equal
cfa-miR-193b	**hsa-miR-193a-3p**	Equal
cfa-miR-193a	**hsa-miR-193a-5p**	Equal
cfa-let-7c	**hsa-let-7a-5p**	Equal
cfa-miR-184	**hsa-miR-184**	Equal
cfa-miR-676	-	Equal
cfa-miR-1306	**hsa-miR-1306-5p**	Equal
cfa-miR-574	**hsa-miR-574-3p**	Equal
cfa-miR-454	**hsa-miR-130a-3p**	Equal
cfa-miR-497	**hsa-miR-15a-5p**	Equal
cfa-miR-152	-	Equal
cfa-miR-204	**hsa-miR-204-5p**	Equal
cfa-miR-30a	**hsa-miR-30a-5p**	Equal
cfa-miR-107	**hsa-miR-103a-3p**	Equal
cfa-miR-197	**hsa-miR-197-3p**	Equal
cfa-miR-1271	**hsa-miR-96-5p**	Equal
cfa-miR-126	**hsa-miR-126-5p**	Equal
cfa-miR-328	**hsa-miR-328**	Equal
cfa-miR-149	**hsa-miR-149-5p**	Equal
cfa-miR-1307	-	Equal
cfa-miR-146a	**hsa-miR-146a-5p**	Equal
cfa-miR-138a	**hsa-miR-138-5p**	Equal
cfa-miR-375	**hsa-miR-375**	Equal
cfa-miR-195	**hsa-miR-15a-5p**	Equal
cfa-miR-660	**hsa-miR-660-5p**	Equal
cfa-miR-218	**hsa-miR-218-5p**	Equal
cfa-miR-23b	**hsa-miR-23a-3p**	Equal
